# Functionalized Collagen/Poly(ethylene glycol) Diacrylate Interpenetrating Network Hydrogel Enhances Beta Pancreatic Cell Sustenance

**DOI:** 10.3390/gels9060496

**Published:** 2023-06-19

**Authors:** Natalia Moreno-Castellanos, Elías Cuartas-Gómez, Oscar Vargas-Ceballos

**Affiliations:** 1 Centro de Cromatografía y Espectrometría de Masas, CROM-MASS, Universidad Industrial de Santander, Cra 27 calle 9, Bucaramanga 680002, Colombia; 2 CICTA Research Group, Department of Basic Sciences, Medicine School, Health Faculty, Universidad Industrial de Santander, Cra 27 calle 9, Bucaramanga 680002, Colombia; 3 GIMAT Research Group, Escuela de Ingeniería Metalúrgica y Ciencia de Materiales, Universidad Industrial de Santander, Cra 27 calle 9, Bucaramanga 680002, Colombia; osavarce@uis.edu.co

**Keywords:** collagen, hydrogel, interpenetrating network, beta pancreatic cells, vascular endothelial factor, biomaterial

## Abstract

Three-dimensional matrices are a new strategy used to tackle type I diabetes, a chronic metabolic disease characterized by the destruction of beta pancreatic cells. Type I collagen is an abundant extracellular matrix (ECM), a component that has been used to support cell growth. However, pure collagen possesses some difficulties, including a low stiffness and strength and a high susceptibility to cell-mediated contraction. Therefore, we developed a collagen hydrogel with a poly (ethylene glycol) diacrylate (PEGDA) interpenetrating network (IPN), functionalized with vascular endothelial growth factor (VEGF) to mimic the pancreatic environment for the sustenance of beta pancreatic cells. We analyzed the physicochemical characteristics of the hydrogels and found that they were successfully synthesized. The mechanical behavior of the hydrogels improved with the addition of VEGF, and the swelling degree and the degradation were stable over time. In addition, it was found that 5 ng/mL VEGF-functionalized collagen/PEGDA IPN hydrogels sustained and enhanced the viability, proliferation, respiratory capacity, and functionality of beta pancreatic cells. Hence, this is a potential candidate for future preclinical evaluation, which may be favorable for diabetes treatment.

## 1. Introduction

Type I diabetes mellitus (T1D) is a chronic metabolic disease characterized by the destruction of pancreatic beta cells [1]. T1D requires life-long insulin therapy; however, several complications are associated with insulin injections, including hypoglycemic events and the persistence of macrovascular complications [2,3]. Therefore, beta cell replacement therapy is a promising treatment for T1D [4]. However, the success of this strategy is affected by a decrease in cell mass due to mechanical stress, a lack of oxygen, vascularization, an immediate blood-mediated immune response (IBMIR), and a lack of islet availability [5]. In this sense, three-dimensional (3D) biomaterials such as hydrogels have been used to improve beta cell viability, thus reducing the number of donor organs needed for transplantation [5,6]. Furthermore, cell encapsulation technology offers an alternative strategy in which a passive barrier separates implanted cells from the hostile immune system [5].

Some studies have reported that extracellular matrix (ECM)-based matrices can sustain islet and beta cell growth and functionality [7,8,9,10,11]. However, these constructs as medical devices should have the ability to house a sufficient number of beta pancreatic cells to fulfill insulin requirements [5]. In addition, after implantation, the construct must be prone to the presence of a vascular network in close proximity [5]. This is important because revascularization ensures an efficient exchange of nutrients, metabolites, and hormones and the maintenance of adequate oxygen tension, which are important for the survival of cells [5,12]. Nonetheless, the use of biomaterials as a protection mechanism against the host immune system is traded for low oxygen levels, leading to low islet survival owing to a suboptimal environment [5]. In addition, it has been reported that biomaterials must provide mechanical protection to cells to retain their native-like morphology [13].

Type I collagen is an ECM protein abundant in many tissues, including the pancreas [14]. Hence, the use of this ECM component in hydrogels as a beta cell supportive biomaterial have been somewhat investigated [7,8,9]. For instance, Weber et al. [15] established a new 3D cell culture system for the generation of an extracellular environment to promote isolated β cell survival and function using a collagen-based matrix. In addition, Llacua et al. [9] reported that collagen supports the in vitro viability and survival of human pancreatic islets. However, pure collagen hydrogels possess several mechanical impairments because they tend to undergo rapid cell-mediated degradation, which can be challenging to control and predict [16].

Several strategies to improve collagen hydrogel stiffness, strength, and resistance to degradation and cell-mediated compaction have been developed using different cell models, including poly(ethylene glycol) diacrylate (PEGDA) [16,17,18]. The integration of this molecule into collagen networks permits the formation of interpenetrating networks (IPNs) [16,19]. IPNs consist of two separate polymers that are crosslinked independently; in this case, collagen is physically crosslinked and PEGDA infiltrates the collagen network, resulting in a collagen/PEGDA IPN hydrogel with a PEGDA network [16,19]. This design has enhanced the properties needed for cell survival [16,17,18]. Additionally, vascular endothelial growth factor (VEGF) is a peptide and powerful molecule involved in various cellular processes, including function and signaling between cells [13,20,21,22,23]. It induces cell proliferation and functionality enhancement and has been added to collagen hydrogels to have neuro-osteoinductive potential [13]. In addition, it has been combined with other molecules for drug delivery, resulting in improved diabetic tissue regeneration and gene delivery [24,25]. However, the inclusion of VEGF in collagen/PEGDA IPN hydrogels to support beta pancreatic cell growth, proliferation, and functionality has been poorly investigated.

In the current study, we developed a collagen hydrogel (2 mg/mL) with a covalently crosslinked PEGDA network to increase the mechanical response by adding VEGF at different concentrations (1, 3, and 5 ng/mL) to evaluate the effect of the degree of functionalization on the hydrogels. We analyzed the physicochemical characteristics of the biomaterials and their rheological behavior in response to deformation. We established the swelling behavior and its degradation over time. Thereafter, we evaluated the effects of the developed hydrogels on the viability, growth, mitochondrial respiration, and functional behavior of beta pancreatic cells. The aim of our study was to develop a collagen hydrogel with a PEGDA IPN functionalized with VEGF directly loaded to mimic the pancreatic environment for the sustenance of beta pancreatic cells as a strategy for diabetes therapy.

## 2. Results and Discussion

### 2.1. Physicochemical Characterization

#### 2.1.1. Fourier Transform Infrared (FTIR) Spectroscopy

**Figure 1:**
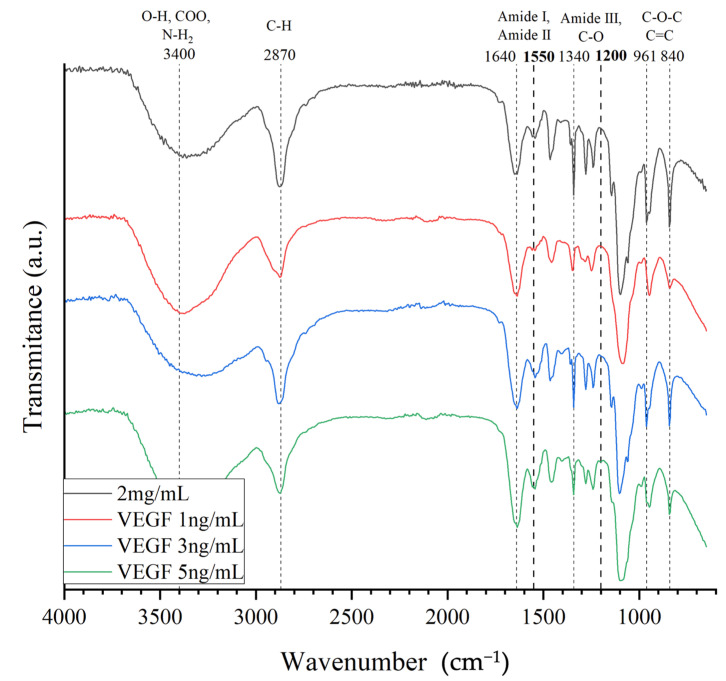
FTIR spectra of acellular collagen hydrogel with PEGDA IPN. Collagen (2 mg/mL) was functionalized with 1, 3, or 5 ng/mL VEGF. The dotted lines with bold numbers correspond to functional groups associated with the VEGF peptide.

FTIR elemental analysis was performed to demonstrate the integration of the basic components into the chemical structure of the functionalized collagen/PEGDA IPN hydrogels (Figure 1). Initially, in all experimental groups, a stretch band at 3000–3600 cm^−1^ positions were observed, representing OH bonds, carbonyl groups, and NH_2_. These bonds are strongly related to amide A of Type I collagen, especially when crosslinked [26,27]. Carbonyl groups present at the same position are usually associated with the formation of collagen peptides. In addition, the OH bonds highlight the absorbed water in the constructs [26,27]. We also observed the stretching of CH and CH_2_ at 2870 cm^−1^, which corresponds to amide B found in natural collagen [27]. It has been demonstrated that collagen exposed to UV irradiation shifts amide A towards lower spectral ranges [28,29]. Nevertheless, these changes were not observed, suggesting that the hydrogen bonds were not destroyed. Therefore, variations in the structural order of collagen were not expected.

At 1640 cm^−1^, bonding between NH and the C=O stretch formed by amide I, which governs the secondary structure of the peptides, was observed. [26,27] Additionally, the position at 1550 cm^−1^ indicates C–N stretching and N–H bending vibrations, which correspond to amide II and are consistent with type I collagen [27,30]. We observed the presence of amide III at 1340 cm^−1^, confirming that the intact triple-helical structure of collagen was well maintained [27,30,31]. Previous analyses have reported that peptide bonds in the collagen structure can be damaged by UV exposure [31,32]; however, we did not find changes or absences in the elemental analysis of the composition of the collagen peptide, suggesting that UV irradiation did not impair collagen crosslinking.

In particular, there was an overlap at 2870 and 1640 cm^−1^, which corresponded to the asymmetric stretching of CH_2_ and the symmetric vibration of C=C groups, respectively, typically found in the PEGDA backbone [33,34]. Nonetheless, the presence of a symmetric vibration at 1720 cm^−1^ is usually associated with the C=O of the acrylate groups in PEGDA [34,35,36]. Moreover, characteristic absorption bands at 1100 and 950 cm^−1^, which are associated with the C–O–C and C–O vibrational modes of the PEGDA backbone, were also observed. This suggests that PEGDA acts as an interpenetrating network (IPN) in the collagen matrix, and PEGDA was successfully integrated. Notably, the photoinitiator aids the polymerization process by the homolytic bond cleavage of radicals upon exposure to UV light [19]. Similarly, the presence of the 1200 cm^−1^ band has been associated with the VEGF peptide, as previously suggested [37]. Furthermore, it can be observed that this peak was more noticeable with increasing concentrations of VEGF peptide to the structure than to the non-functionalized hydrogel. Hence, using FTIR spectroscopy, we demonstrated that the components of the hydrogel, collagen with PEGDA and VEGF, were successfully incorporated.

#### 2.1.2. Scanning Electron Microscopy (SEM)

**Figure 2:**
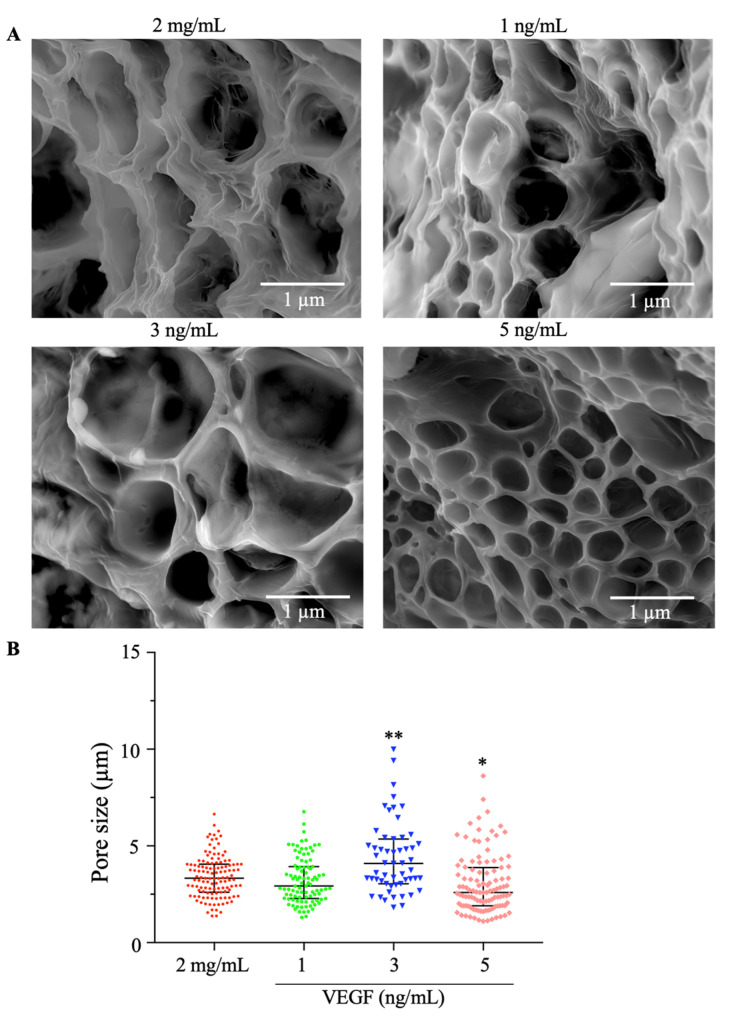
Microstructural behavior of collagen/PEGDA IPN hydrogels. (**A**) Scanning electron microscopy micrographs of functionalized acellular collagen/PEGDA IPN hydrogels (scale bar: 1 µm; 15,000×). (**B**) Pore size of hydrogels (μm) (n ≥ 3, median with interquartile range, Kruskal–Wallis, *p* < 0.05, Dunn’s multiple comparisons test * *p* < 0.05, ** *p* < 0.01).

To evaluate the microstructural modification of the functionalized collagen/PEGDA IPN hydrogels, scanning electron microscopy (SEM) was performed, as shown in Figure 2. A microporous structure with irregular architecture was formed. The structures found in the current study were consistent with those of other hydrogels in which PEGDA was added [36,38]. In addition, we measured these pores and observed that they were distributed across the constructs. The pore size of the 2 mg/mL hydrogels varied from 1.375 to 6.640 μm, with a mean of 3.418 μm, which was similar to the condition of 1 ng/mL VEGF because the pore sizes ranged from 1.302 to 6.774 μm. However, we found that 3 ng/mL (*p* = 0.0021) and 5 ng/mL (*p* = 0.0163) had a significantly larger pore distribution than the 2 mg/mL concentration. These pores varied for the 3 ng/mL condition from 1.818 to 9.993 μm and for the 5 ng/mL condition from 1.110 to 8.615 μm.

This analysis suggests that high concentrations of VEGF induced structural changes in the microstructure of the collagen/PEGDA hydrogel because larger pores were found under the 3 and 5 ng/mL experimental conditions. Our findings are consistent with some reports in which VEGF-loaded structures induced the presence of pores [22]. These structures have been reported to facilitate communication between cells and the transmission of nutrients [36,38]. In addition, the structures found in our collagen/PEGDA IPN hydrogels are consistent with mesopores (2–50 μm), which are expected to promote cell adhesion and insulin secretion and modulate cell proliferation [12]. These structures play an essential role in cellular behavior because they allow the growth of blood vessels, deposition of ECM components, cell growth, and transport of nutrients and oxygen throughout the polymeric network [11,12]. Moreover, the presence of pores has been reported to enhance the mechanical properties and stability of hydrogels [7,39,40].

#### 2.1.3. Rheological Behavior

**Figure 3:**
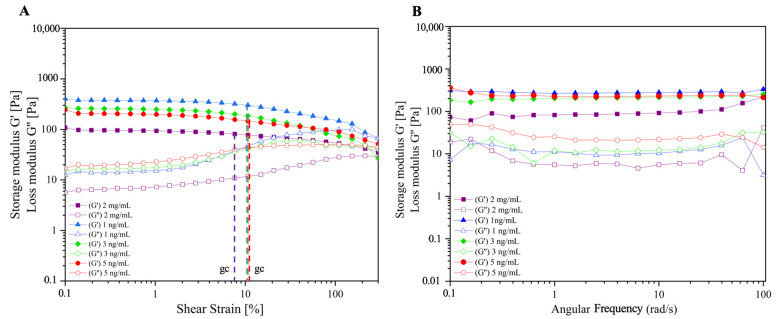
Rheological analysis of acellular collagen hydrogel. (**A**) Amplitude and (**B**) frequency sweeps of 2 mg/mL of collagen functionalized with 1, 3, and 5 ng/mL of VEGF.

We performed a rheological analysis in order to evaluate the storage (𝐺’) and loss (𝐺’’) moduli of functionalized collagen/PEGDA IPN hydrogels as a function of the shear stress applied (Figure 3). It is seen that the storage/elastic modulus of all the conditions is higher than the loss/viscous modulus (𝐺’ > 𝐺’’) (Figure 3A). Our hydrogels exhibited rigidity and stability and behaved as gel-like structures [41]. This can be explained by the infiltration of PEGDA into the collagen polymer network, forming a sequential IPN. PEGDA has been used to increase the stability, stiffness, and strength of single-component networks [16,17,33,38]. Furthermore, PEGDA acts synergistically rather than simply as an additive to tailor the mechanical properties of the hydrogels [16].

Interestingly, VEGF-loaded hydrogels exhibited a higher elastic behavior than non-functionalized constructs. However, increasing the VEGF content did not affect the stiffness of the functionalized hydrogels. This may be explained by the direct loading method used to functionalize the constructs. Hence, the hydrogel becomes crowded, and the VEGF–collagen interaction increases; thus, the elastic behavior increases [13]. However, it has been found that oversaturation of the VEGF peptide in a hydrogel structure results in a decrease in the mechanical stability of the hydrogels [21]. Therefore, it is desirable to optimize the VEGF concentration to sustain this stability. In addition, steric hindrance and hydrogel mesh size may be other factors that influence the advanced elastic behavior of functionalized hydrogels [13], which may be associated with the porous structure observed in the SEM results, as discussed in the previous section.

All hydrogels presented a linear viscoelastic region (LVE), which expresses the independence between 𝐺’ and 𝐺’’ of the deformation that occurs. This is directly related to the stable and structured shapes of the hydrogels [41,42]. The observation of the critical shear deformation, also known as gc, can also be determined as the value of maximum deformation in which the value of 𝐺’ remains constant [42]. This is obtained by the analysis of the plateau of the 𝐺’-curve combine with the 𝐺’-values and when they are beginning to show a deviation of 10% from the previous constant values [41,42,43]. As shown in Figure 3A (dashed lines), all VEGF-loaded hydrogels had a greater gc (10.7%) than non-functionalized hydrogels (7.65%), supporting the idea that VEGF positively affects the structural stability of the hydrogel. This is in agreement with Yin et al. [21], who reported that VEGF concentrations of 1–10 ng/mL positively influenced the mechanical stability of the hydrogels.

In addition, Figure 3B reveals the oscillatory tests where the 𝐺’ and 𝐺’’ are compared to the angular frequency applied. In all cases, we observed the viscoelastic behavior of the hydrogels, and the storage modulus prevailed over the loss modulus. This confirmed the stability of the hydrogels as a function of the applied frequency. In addition, the behavior of glassy solids is established with a typical oscillatory response for a gel-like material with a covalent polymer network formed by crosslinking PEGDA chains with a physically crosslinked collagen network [16,42,44]. These results confirm that the variation in the composition of hydrogels affects their deformation and flow, resulting in tunable materials [44]. This is advantageous for implantation applications because it resists shear stress at the in vivo site used for grafting.

#### 2.1.4. Swelling and Degradation Rate

**Figure 4:**
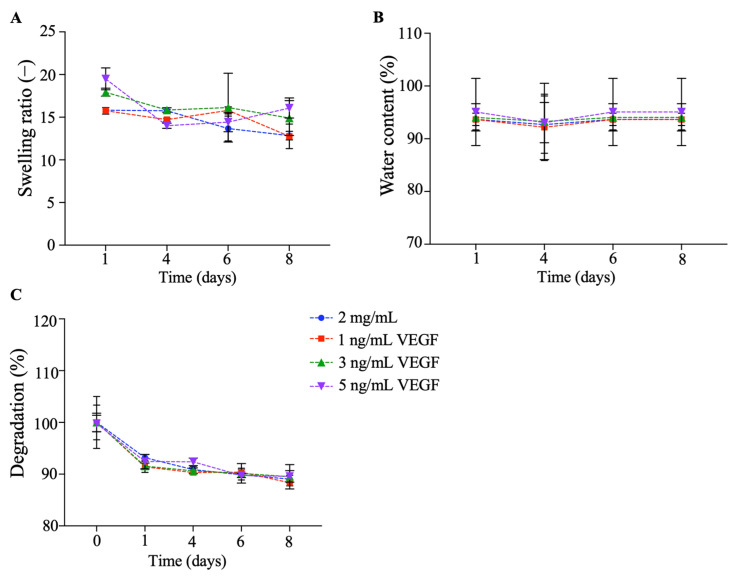
Hydrogel stability over time: (**A**) swelling (−); (**B**) water content; and (**C**) degradation (%) performance of the functionalized collagen/PEGDA IPN hydrogels (n ≥ 3, mean ± SEM; one-way ANOVA, *p* > 0.05).

To determine the water absorbency capacity and in vitro degradability over time, the swelling ratio and degradation percentage were assessed (Figure 4). On day 1 (Figure 4A), the swelling ratios of the 2 mg/mL and 1, 3, and 5 ng/mL VEGF collagen/PEGDA IPN hydrogels were 15.813 ± 0.274, 15.753 ± 0.379, 17.933 ± 0.464, and 19.497 ± 0.288, respectively. We did not find statistically significant differences between the groups (*p* > 0.05), but there was a tendency for the 3 and 5 ng/mL VEGF conditions to have a greater water absorption capacity than the 2 mg/mL and 1 ng/mL VEGF conditions. This characteristic is a key parameter because a high degree of swelling makes them excellent candidates for highly biocompatible materials [44].

After eight days of culture, we found that the 5 ng/mL VEGF condition had a high swelling ratio (16.077 ± 1.178). However, there was a decrease (*p* > 0.05) in the swelling ratio for the 2 mg/mL, 1 ng/mL, and 3 ng/mL VEGF hydrogels, with mean values of 12.847 ± 0.500, 12.767 ± 1.437, and 14.907 ± 2.034, respectively. These results are consistent with those of Munoz-Pinto et al. [16], who found that collagen/PEGDA IPN hydrogels had the same degree of swelling as our hydrogels. However, we discovered that the 5 ng/mL VEGF condition exhibited a tendency to sustain absorbed water over time compared to the other conditions. These results confirm the capacity of the functionalized material (5 ng/mL of VEGF) to maintain sufficiently high swelling values for its application as a cytocompatible biomaterial [44]. This rich water content provides a physical environment similar to that of native tissue, thus offering an intimate environment for cells to reside and grow [45].

To verify this, we analyzed the water content percentage (Figure 4B) and found that the content on day 1 was over 90% for all conditions. Similarly, the hydrogel maintained this percentage after eight days of incubation, and no statistical differences were found among the groups (*p* > 0.05). However, it is important to highlight that we found that the 5 ng/mL hydrogel had a percentage of over 95%, whereas the other conditions ranged from 90 to 93%. This is consistent with previous findings, where collagen hydrogels were found to retain a large water volume owing to the high hydrophilicity of the collagen peptide [13,16,17]. Furthermore, this suggests that the osmotic pressure between the hydrogel network and the external solution did not significantly prevent water molecules from penetrating the hydrogels [46]. Hence, we suggest that the high water content of our hydrogels supports the encapsulation of hydrophilic molecules such as VEGF to avoid denaturation and aggregation [45].

In contrast, we evaluated the percentage of in vitro degradation over time (Figure 4C). All hydrogels started with a mass of 100% and eventually started to lose mass. After eight days of incubation, the remaining masses were 89.057 ± 0.737, 88.300 ± 0.612, 89.503 ± 2.340, and 89.580 ± 1.099% for the 2 mg/mL and 1, 3, and 5 ng/mL VEGF conditions, respectively. This is in agreement with previous findings, where after 14 days of culture, the collagen/PEGDA IPN hydrogel did not lose its shape and exhibited low degradation behavior [16]. The evaluation of degradation is important in the design of biomaterials for tissue engineering applications. The in vivo degradation rate properties and stability in a living body are determined by the degradation behavior of the biomaterial [5]. We suggest that our functionalized hydrogels did not lose their capability to absorb water and that the mass loss was not drastically reduced over time.

Additionally, we evaluated the release profile of VEGF as a function of time (Figure S1). For 3 and 5 ng/mL of VEGF, we observed that the protein was delivered gradually during the days evaluated, reaching a cumulative release of 50% in four days. We found that 1 ng/mL of VEGF did not match this release rate, with a less than 40% release on day 4. This may be explained by the small quantity of protein loaded into the hydrogel [47]. These findings are consistent with previous reports where the direct loading of growth factor proteins, including VEGF, into collagen hydrogels resulted in a high release over time [48,49,50]. This would be favorable for the vascularization process in situ, which is required for successful graft implantation and survival of pancreatic cells [5].

The main mechanisms mediating the release of this protein are the swelling and degradation rates of the hydrogels, as previously suggested [13,51,52]. A swollen hydrogel would allow molecules to diffuse outside the construct, coupled with the steady degradation rate of the biomaterial, resulting in a beneficial release profile of proteins, which is consistent with previous findings [48,49].

### 2.2. Biocompatibility Assays

#### 2.2.1. Cell Viability and Proliferation

**Figure 5:**
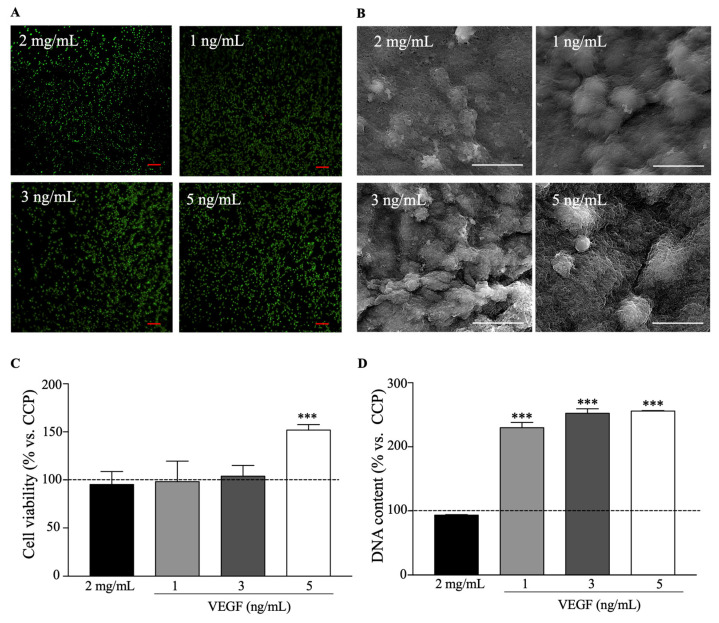
Biocompatibility assays of functionalized hydrogels with encapsulated cells: (**A**) Live and dead assay of encapsulated beta pancreatic cells in VEGF-functionalized collagen (2 mg/mL)/PEGDA IPN hydrogels after 48 h (scale bar in red: 200 µm); (**B**) Scanning electron microscopy (SEM) images of cells encapsulated in collagen/PEGDA IPN matrix (scale bar: 5 µm); (**C**) Viability of encapsulated β pancreatic cells in VEGF-functionalized collagen hydrogel crosslinked with PEGDA IPN after 48 h. The combination of 2 mg/mL and 5 ng/mL of VEGF resulted in the highest cell viability; (**D**) DNA content of encapsulated beta pancreatic cells in VEGF-functionalized collagen/PEGDA IPN hydrogel after 72 h (n ≥ 3, one-way ANOVA, *p* < 0.05, *** *p* < 0.001 vs. cell culture plate.

To evaluate the viability and proliferation of encapsulated beta pancreatic cells in collagen/PEGDA IPN hydrogels, we performed live/dead, MTT, and PicoGreen assays (Figure 5). The live/dead assay uses fluorescence microscopy with fluorophores to indicate whether the integrity of the membrane cells is altered (red: dead) or whether they are metabolically active (green: live). No substantial alterations in cell membrane integrity were observed under any of these conditions (Figure 5A). We observed the presence of a large number of live cells throughout each condition, supporting the hypothesis that this biomaterial is not cytotoxic and supports beta pancreatic cells. This is in agreement with previous studies that used different encapsulated cell models in collagen/PEGDA IPN hydrogels, where their viability was conserved [16,17,53].

In addition, we performed SEM to analyze the distribution of beta pancreatic cells and their interactions with the collagen/PEGDA IPN matrix. As shown in Figure 5B, cells were embedded in the collagen/PEGDA IPN network. This analysis suggests that the cells are close to each other and are sustained by the non-functionalized and functionalized hydrogel matrix.

Additionally, we confirmed the ability of these hydrogels to support live beta pancreatic cells using the MTT assay (Figure 5C). After 48 h of incubation, the mean cell viability values at 2 mg/mL, 1 ng/mL, and 3 ng/mL were 95.287 ± 13.442 (*p* > 0.9999), 98.204 ± 21.299 (*p* > 0.9999), and 103.907 ± 11.207% (*p* > 0.9999), respectively, compared to CCP. However, after the same time period, 5 ng/mL of VEGF showed a significant (*p* = 0.0028) increase in cell viability (151.957 ± 5.582%) compared to CCP, suggesting that cell growth was stimulated. This was confirmed by a cell proliferation assay (Figure 5D) after 72 h of incubation. We found that 1, 3, and 5 ng/mL of VEGF had significantly higher DNA concentrations than CCP, with mean values of 230.000 ± 8.155 (*p* < 0.001), 252.300 ± 7.093 (*p* < 0.001), and 256.000 ± 0.750% (*p* < 0.001), respectively.

Previous reports have found that collagen/PEGDA IPNs can sustain cell viability, but cells do not proliferate [16,17]. A possible explanation for this is that these studies developed collagen hydrogels at a higher concentration (3 mg/mL), which translates into a stiffer material. Stiff hydrogels influence cell behavior through intracellular signals, which reduce cell spreading and growth [53,54]. Moreover, they reported that PEGDA-containing hydrogels have slow degradation rates and differ from the nanoscale crosslinks of pure collagen hydrogels [16]. However, we found that the optimal concentration was 2 mg/mL, without drastically losing stability over time, which confirmed that this was the ideal baseline concentration; therefore, it was functionalized. In this study, we developed tunable, functionalized collagen/PEGDA IPN hydrogels that can support live cells and promote cell proliferation.

The increased proliferation of beta pancreatic cells in functionalized hydrogels may be due to the mesopores that are influenced by VEGF, as shown in the SEM analysis in Section 2.1.2. This is in agreement with previous analyses, which stated that pores have a profound impact on the fate of encapsulated cells [54]. The degree of functionalization with VEGF enhanced the cell viability and did not alter the stability or elasticity of the hydrogels in a concentration-dependent manner [55]. Indeed, there was a decrease in stress relaxation in the collagen/PEGDA IPN matrix at 2 mg/mL compared to that at higher collagen concentrations, as reported previously [16,17]. However, this decrease reduces cell-mediated matrix compaction, resulting in cell proliferation [16,56].

Another possible explanation may be the presence of VEGF, as other reports have stated that this growth factor helps the formation of islets, and, thus, cell proliferation [57,58]. Additionally, VEGF binds to several tyrosine kinase receptors to promote vascularization and deliver nutrients to cells, whereas biomaterials protect these cells from the immune response [5,57]. However, high VEGF concentrations have been described as deleterious for the survival of beta pancreatic cells [57]; hence, optimal concentrations must be considered. Thus, we suggest that VEGF (at concentrations of 1, 3, and 5 ng/mL) in collagen/PEGDA IPN hydrogels has positive effects on cell viability and proliferation.

#### 2.2.2. Oxygen Flow

**Figure 6:**
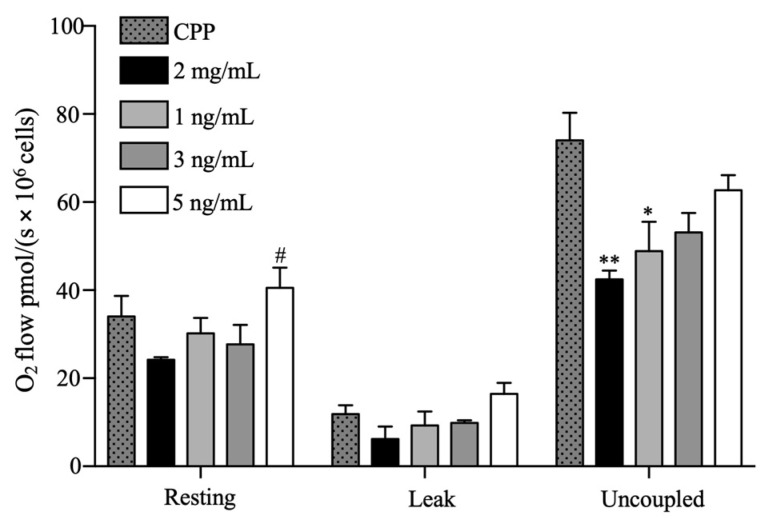
The effect on oxygen flow in all experimental groups was expressed as O_2_ flow pmol/(s × 10^6^ cells). One-way ANOVA, n ≥ 3, * *p* < 0.05, ** *p* < 0.01, vs. CCP, # *p* < 0.05 vs. 2 mg/mL.

To evaluate oxygen flow in beta pancreatic cells and the influence of hydrogels on these cells, we performed high-resolution respirometry (Figure 6). After 24 h of incubation, basal respiration was maintained in almost all the cases (*p* > 0.05). However, basal respiration was improved at a concentration of 5 ng/mL of VEGF, with a mean value of 40.577 ± 4.586 pmol/(s × 10^6^ cells) (*p* = 0.0183) compared with the 2 mg/mL condition.

No significant differences were observed in the leak state between the experimental groups. In this respiratory state, we found that 2 mg/mL and 1, 3, and 5 ng/mL of VEGF had mean oxygen flow values of 6.184 ± 2.849 (*p* = 0.4390), 9.254 ± 3.216 (*p* = 0.9204), 9.889 ± 0.571 (*p* = 0.9688), and 16.418 ± 2.517 (*p* = 0.6436) pmol/(s × 10^6^ cells), respectively, compared to CCP.

In the state of maximal respiration capacity in the uncoupled state, we found that 2 mg/mL and 1 ng/mL of VEGF significantly decreased oxygen consumption with mean values of 42.487 ± 1.932 (*p* = 0.0068) and 48.888 ± 6.669 (*p* = 0.0278) pmol/(s × 10^6^ cells), respectively, compared to CCP. Similarly, under 3 and 5 ng/mL VEGF conditions, the uncoupled state was not affected. The oxygen flow in the uncoupled state for 3 ng/mL was 53.157 ± 4.425 (*p* = 0.0729) and for 5 ng/mL was 62.737 ± 3.383 (*p* = 0.5004) pmol/(s × 10^6^ cells) compared to CCP.

Cumulatively, our results suggest that 5 ng/mL of VEGF induced a more stable respiratory capacity than the other experimental groups. This may be explained by the stimulatory effect of VEGF on the expression of a cluster of nuclear-encoded mitochondrial genes in different cell types, suggesting a role of VEGF in the upregulation of mitochondrial biogenesis [59]. Specifically, the presence of VEGF results in the activation of Akt3, which controls the nuclear localization of a master regulator of mitochondrial biogenesis, thereby enhancing respiratory capacity [59,60]. However, our results suggest that small concentrations of VEGF in the 3D matrix are not sufficient to sustain the mitochondrial respiratory capacity of beta pancreatic cells.

Another possible explanation is that the collagen/PEGDA IPN structure may have affected the encapsulated cells. Our SEM analysis suggests that VEGF induces larger pore sizes, which facilitates the transport of nutrients, including oxygen, which means that more oxygen is available for consumption. Previous analyses have reported that oxygen is readily diffusible in collagen 3D constructs [61]. Therefore, we suggest that the functionalized hydrogel matrix structure may enhance the mitochondrial oxygen consumption of beta pancreatic cells.

#### 2.2.3. Functional Behavior

**Figure 7:**
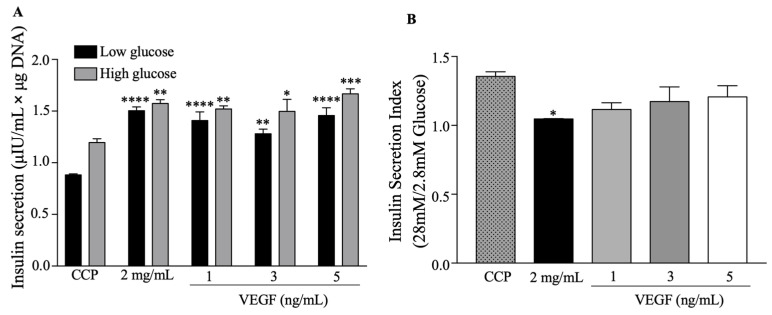
Functional behavior of beta pancreatic cells in response to low and high glucose concentrations in collagen/PEGDA IPN hydrogels: (**A**) insulin secretion by encapsulated pancreatic beta cells is expressed as μIU/mL×μg DNA; (**B**) insulin secretion index. One-way ANOVA, *p* < 0.05, n ≥ 3, * *p* < 0.05, ** *p* < 0.01, *** *p* < 0.001, **** *p* < 0.0001 vs. CCP. Data points represent the mean ± SEM.

We performed glucose-stimulated insulin secretion analysis (GSIS) to evaluate the effect of the functionalized collagen/PEGDA IPN hydrogels on the functional behavior of beta pancreatic cells (Figure 7). After 48 h of culture, the cells were exposed to low glucose levels (Figure 7A). At 2 mg/mL and 1, 3, and 5 ng/mL of VEGF, insulin secretion was significantly increased with mean values of 1.503 ± 0.037 (*p* < 0.0001), 1.409 ± 0.083 (*p* < 0.0001), 1.281 ± 0.046 (*p* = 0.0011), and 1.457 ± 0.075 μIU/mL × μg DNA (*p* < 0.0001), respectively, compared to CCP. Similarly, we stimulated the encapsulated cells with a high glucose concentration. At this concentration, all experimental groups showed an increase in insulin secretion, with mean values of 1.574 ± 0.037 (*p* = 0.0019) for 2 mg/mL, 1.520 ± 0.029 for 1 ng/mL (*p* = 0.0082), 1.497 ± 0.117 for 3 ng/mL (*p* = 0.0151), and 1.687 ± 0.048 for 5 ng/mL (*p* = 0.0002), for μIU/mL × μg DNA, respectively.

However, we found that the insulin secretion index (Figure 7B) (ratio between the insulin secreted at 28 mM and 2.8 mM glucose concentrations) for 2 mg/mL was significantly reduced, with a mean value of 1.047 ± 0.002 (*p* = 0.0246) when compared with CCP. Nevertheless, the secretion index was sustained in the functionalized collagen hydrogels (1, 3, and 5 ng/mL).

A possible explanation for the increased insulin secretion by encapsulated beta pancreatic cells may be cell–matrix interactions [15,62]. It has been suggested that this interaction improves beta pancreatic cell survival and insulin secretion, and the whole collagen protein structure and multiple cell surface receptors increase cell survival and function [15]. This may be explained by F-actin remodeling, which has been shown to be necessary for insulin secretion after high glucose induction [15]. Some studies have reported that cortical F-actin must be rearranged to improve insulin secretion [13,58,63]. Therefore, the interaction between the 3D matrix structure and cytoskeleton may be involved in improved insulin secretion in encapsulated beta pancreatic cells. In addition, baseline insulin secretion was strongly increased in the hydrogels. Changes in the microenvironment from 2D to 3D can explain the modifications in insulin secretion after low-glucose stimulation; however, this must be studied further [64].

Our findings suggest that functionalized collagen/PEGDA IPN hydrogels can sustain and increase insulin secretion by pancreatic beta cells. Additionally, cells encapsulated in the functionalized hydrogels responded to high-glucose stimulation. Nonetheless, we suggest that if cells are in a non-functionalized environment they will not be stimulated by high glucose concentrations, thereby losing their responsiveness. Our findings are in agreement with previous reports stating that VEGF enhances insulin secretion [65]. However, the exact mechanism by which VEGF stimulates insulin secretion in encapsulated pancreatic beta cells is poorly understood and requires further investigation.

## 3. Conclusions

In conclusion, we developed collagen/PEGDA IPN hydrogels with different VEGF concentrations (1, 3, and 5 ng/mL). Physicochemical analysis of the hydrogels confirmed the presence of the characteristic elemental groups of type I collagen, along with the presence of PEGDA and VEGF. In addition, we determined that hydrogels with PEGDA and VEGF exhibited conformational changes in the biomaterial matrix, which may have influenced the stable water retention and weight loss. Furthermore, we found that the hydrogels were tunable and mechanically stable with a gel-like solid structure. We performed biocompatibility, cell respiration, and functionality assays, which helped us to confirm that almost all functionalized hydrogels were able to sustain or improve these parameters. However, our results cumulatively indicated that the 5 ng/mL VEGF treatment resulted in an increased cell viability, improved proliferation, sustained oxygen consumption, and enhanced insulin secretion. To the best of our knowledge, this is the first study to successfully synthesize a functionalized collagen/PEGDA IPN hydrogel that supports and induces the growth of functional beta pancreatic cells without affecting mitochondrial respiration. This is a promising biomaterial that may be used in future preclinical studies on diabetes treatment in clinical beta cell replacement therapy.

## 4. Materials and Methods

### 4.1. Cell Culture

The BRIN-BD11 (ECACC 10033003) cell line was cultured at 37 °C-5% CO_2_, according to the manufacturer’s guidelines. The cells were maintained in a RPMI-1640 medium (Sigma-Aldrich, St. Louis, MO, USA) supplemented with 10% fetal bovine serum (FBS) (Sigma-Aldrich, St. Louis, MO, USA), 2 mM of Glutamine (Sigma Aldrich, St. Louis, MO, USA), and penicillin/streptomycin/amphotericin B 1% (Sigma Aldrich, St. Louis, MO, USA). The medium was changed every three days and subcultured with trypsin/EDTA (Sigma Aldrich, St. Louis, MO, USA), at a density of 2 × 10^5^ cells/cm^2^.

### 4.2. Interpenetrating Network (IPN) Hydrogel Fabrication

Collagen/PEGDA IPN hydrogels were fabricated at a collagen concentration of 2 mg/mL and functionalized with vascular endothelial growth factor (VEGF) at different concentrations [16,23]. In brief, type I collagen (Sigma Aldrich, St. Louis, MO, USA) was neutralized. VEGF (Sigma Aldrich, St. Louis, MO, USA) at concentrations of 1, 3, and 5 ng/mL was directly loaded onto the gel-forming solution. The cells were mixed with the solution at a concentration of 1 × 10^6^ cells/mL and microdispensed in a 96 well-plate. After 5 min of incubation at 37 °C, the hydrogels were treated with PEGDA 10% (molecular weight = 20.0 kDa; Sigma Aldrich, St. Louis, MO, USA) and photoinitiator 0.1% (Sigma Aldrich, St. Louis, MO, USA) solutions and allowed to infiltrate the collagen network for 30 min at 37 °C. PEGDA within the collagen network was crosslinked by 5 min of exposure to longwave UV light (365 nm, Spectroline, Melville, NY, USA), and an interpenetrating network (IPN) was formed. The collagen/PEGDA IPN hydrogels were incubated in the culture medium at 37 °C and 5% CO_2_.

### 4.3. Physicochemical Characterization

#### 4.3.1. Fourier Transform Infrared (FTIR) Spectroscopy

FTIR spectroscopy was performed to analyze the functional chemical groups and successful inclusion of components in the developed hydrogels [12]. The dehydrated functionalized collagen hydrogel was placed on an Agilent Cary 630 FTIR spectrometer (Agilent, Santa Clara, CA, USA). All spectra were recorded in the spectral range of 4000–650 cm^−1^ with a resolution of 4 cm^−1^ and eight scans. The spectra obtained were plotted using OriginLab Pro Software Version 9.8.5 (Northampton, MA, USA).

#### 4.3.2. Scanning Electron Microscopy (SEM)

Morphological features of the functionalized collagen/IPN hydrogels with and without cells were analyzed by scanning electron microscopy (SEM). Briefly, the IPN hydrogels were rinsed and fixed in 4% paraformaldehyde (Sigma-Aldrich, St. Louis, MO, USA). Louis, MO) and 2.5% glutaraldehyde (Sigma-Aldrich, St. Louis, MO, USA). They were treated with an increasing series of ethanol concentrations (70, 80, 90, 95, and 100%) (Sigma Aldrich, St. Louis, MO, USA) and hexamethyldisilazane (1:4, 1:3, 1:2, and 1:1; Sigma Aldrich, St. Louis, MO, USA).Samples were sputter-coated, and micrographs were acquired using a Field Emission Gun (FEG) QUANTA FEG 650 with a secondary electron detector (FEI Company, Hillsboro, OR, USA) at an accelerating voltage of 15 kV and magnifications of 6000 × and 15,000×.

#### 4.3.3. Swelling and Degradation Tests

Swelling and degradation tests were performed to assess the capacity of the collagen/PEGDA IPNs to absorb water and their stability over time, respectively. These were used as indicators of the degree of infiltration within the collagen networks and the effect of VEGF on the hydrogels [12,46,66]. Hydrogels were weighed using an analytical balance (Ohaus Pioneer, Ohaus Corporation, Parsippany, NJ, USA) to obtain the initial weight (m_i_), and incubated for 8 days at 37 °C in media cultures. The final swollen mass (m_s_) was weighed and dehydrated to determine the final dry mass (m_d_). The mass swelling ratio (q) was calculated as follows for each condition (1):

q = m_s_/m_d_ (1)

The water content (%) is defined by Equation (2):

Water content (%) = (m_s_ − m_d_)/m_s_ × 100 (2)

The degradation test was performed using Equation (3):

Degradation% = ((m_i_ − m_d_)/m_i_) × 100 (3)

#### 4.3.4. Rheological Analysis

Rheological behavior was analyzed to determine the deformation and flow of the hydrogel. This analysis was performed using an MCR 302 Anton-Paar Rheometer (Anton-Paar, Graz, Austria) with 20 mm diameter parallel plates and a Peltier system for temperature control (37 °C). Amplitude sweeps tests were performed, and a shear strain of 0.1–300%, angular frequency of 10 rad/s, and frequency sweeps of deformation of 0.1% for all samples were applied to determine the storage (𝐺’) and loss moduli (𝐺’’) of the samples.

#### 4.3.5. VEGF Release Profile

An in vitro release study was performed to evaluate VEGF release. VEGF peptide contains tryptophan, tyrosine, and cysteine residues, whose ability to absorb light at 280 nm is commonly used to determine the concentration of proteins in solution [67]. Hence, we measured the protein concentration at 280 nm using a NanoDrop™ One/OneC Microvolume UV-Vis Spectrophotometer (Thermo Fisher Scientific, Wilmington, DE, USA) as suggested before [68,69,70]. Briefly, the functionalized VEGF collagen/IPN hydrogels were incubated for 8 days with 1 mL of PBS 1× (pH 7.0) at 37 °C. PBS 1× was used as a blank, and the supernatant was measured at specific time points. The release profile was expressed as cumulative release (%) as a function of time (days). The fraction of VEGF released was calculated by dividing the cumulative release by the initial loading amount.

### 4.4. Biocompatibility Assays

#### 4.4.1. Cell Viability

To assess the effect of functionalized collagen/IPN hydrogels on the cell viability of pancreatic beta cells, we performed 3-(4,5-dimethylthiazol-2-yl)2,5-diphenyltetrazolium bromide (MTT, Sigma Aldrich, St. Louis, MO, USA) and live/dead assays [42]. Briefly, the encapsulated cells were treated with an MTT reagent at a concentration of 5 mg/mL after 49 h of culture and incubated for 5 h at 37 °C in a 5% CO_2_ atmosphere. The constructs were rinsed with PBS 1× and DMSO was added to dilute the formazan crystals formed. The absorbance at 570 nm was measured using a Multiskan GO spectrophotometer (Thermo Fisher Scientific, Wilmington, DE, USA). The quantitative findings were microscopically confirmed using the live/dead assay with Calcein AM and ethidium homodimer-1 (Thermo Fisher Scientific, Wilmington, DE, USA) and incubated for 45 min at room temperature. Fluorescence imaging was performed using a fluorescence microscope Olympus BX53 (Olympus America Inc., Melville, NY, USA). Images were processed using the ImageJ software (NIH, Bethesda, MD, USA).

#### 4.4.2. Cell Proliferation

To evaluate the effect of the hydrogels developed on cell proliferation, DNA content was measured using the Quant-iT™ PicoGreen^®^ dsDNA Kit (Invitrogen, Thermo Fisher Scientific, Wilmington, DE, USA) [42]. Briefly, encapsulated cells were collected and rinsed with PBS 1× after 72 h of incubation. The samples were vortexed for 30 min to release the DNA, frozen, thawed on ice, and homogenized for 10–15 min. The obtained DNA was mixed with a DNA-binding fluorescent dye solution and a DNA standard was used to interpolate the DNA content. Fluorescence measurements were performed using Fluoroskan Ascent (Thermo Fisher Scientific, Wilmington, DE, USA) at excitation and emission wavelengths of 480 and 520 nm, respectively.

#### 4.4.3. Oxygen Uptake

The mitochondrial respiratory capacity of the hydrogels was analyzed using a high-resolution Oxygraph-2K spectrometer (Oroboros Instruments, Innsbruck, Austria). Cells encapsulated in hydrogels at a concentration of 2×10^6^ cells/mL for each condition were incubated for 24 h and then placed in a chamber with the culture medium at 37 °C under gentle agitation (350 rpm). The oxygen flow was evaluated at different respiratory states: basal, leak, and uncoupled [42]. Basal refers to oxygen consumption without the addition of inhibitors or uncouplers. A leak state was achieved by adding 2.5 μM of oligomycin and an uncoupled state with the addition of 1 μM FCCP. These measurements were corrected after subtracting non-mitochondrial respiration after the addition of 2.5 μM of rotenone and 2.5 μM of antimycin. The results were obtained as oxygen flow per cell (pmol/(seg × 10^6^ cells)) and expressed as relative values compared to cell culture plastic (CCP).

#### 4.4.4. Functional Behavior

To assess beta cell functionality, a static glucose-stimulated insulin secretion (GSIS) assay was performed [42]. Briefly, encapsulated cells in collagen/IPN hydrogels were cultured for 48 h and were treated with a low-glucose (LG, 2.8 mM) and high-glucose (HG, 28 mM) Krebs–Ringer Bicarbonate (KRBH) buffer (125 mM of NaCl, 3 mM of KCl, 1.2 mM of CaCl_2_, 1.2 mM of MgSO_4_, 1 mM of NaH_2_PO_4_, 22 mM of NaHCO_3_, 10 mM of HEPES, and 0.1% BSA (Sigma Aldrich, St. Louis, MO, USA)). The supernatant from each condition after exposure was collected and insulin secretion was measured using a Rat Insulin ELISA kit (Invitrogen, Thermo Scientific, Wilmington, DE, USA). Values were normalized to DNA content (μg/mL) using the Quant-iT PicoGreen assay. Finally, the stimulation index (SI) was calculated as the ratio of high- to low-glucose insulin secretion.

### 4.5. Statistical Analysis

Data are presented as the mean ± standard error of the mean (SEM). Statistical analyses were performed using GraphPad Prism, Version 7 (GraphPad Software, Inc., La Jolla, CA, USA). One-way analysis of variance (ANOVA) was performed followed by a post hoc Tukey’s test or Fisher’s LSD, where *p* < 0.05 was considered significant (* *p* < 0.05, ** *p* < 0.01, *** *p* < 0.001, **** *p* < 0.0001). Each experiment was repeated at least three times.

## Data Availability

Data supporting the findings of this study are available to the corresponding author upon request. The data are not publicly available because of privacy or ethical restrictions.

## Supplementary Materials

The following supporting information can be downloaded at: https://www.mdpi.com/article/10.3390/gels9060496/s1, Figure S1: VEGF cumulative release profile as function of time.
