# Supplementary material for: Functionalized Collagen/Poly(ethylene glycol) Diacrylate Interpenetrating Network Hydrogel Enhances Beta Pancreatic Cell Sustenance
Source: Gels. 2023 Jun 19;9(6):496. doi: 10.3390/gels9060496 (PMC10298015; doi:10.3390/gels9060496)
Supplement: Supplementary file 1 [file gels-09-00496-s001.zip › gels-2451868-supplementary.pdf]

Functionalized collagen/poly(ethylene glycol) diacrylate inter-penetrating network hydrogel enhances beta-pancreatic cell sustenance

Natalia Moreno-Castellanos*[1], Elías Cuartas-Gómez[2], Oscar Vargas-Ceballos[3]

[1] Centro de Cromatografía y Espectrometría de Masas, CROM-MASS, Universidad Industrial de Santan-der, Cra 27 calle 9, Bucaramanga 680002, Colombia
[2] CICTA Research Group, Department of Basic Sciences, Medicine School, Health Faculty, Universidad
Industrial de Santander, Cra 27 calle 9, Bucaramanga 680002, Colombia
[3] GIMAT Research group, Escuela de Ingeniería Metalúrgica y Ciencia de Materiales, Universidad Indus-trial de Santander, Cra 27 calle 9, Bucaramanga 680002, Colombia

*Correspondence: nrmorcas@uis.edu.co

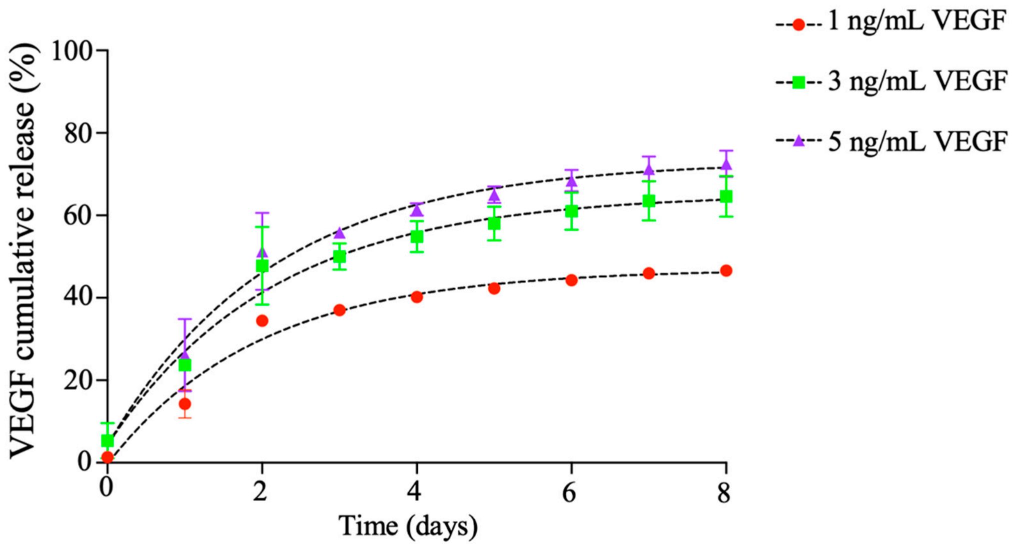

Figure S1: VEGF cumulative release profile as function of time. The *in vitro* release curve from VEGF (directly loaded) functionalized collagen/PEGDA IPN hydrogels in PBS at 37 ºC expressed as a function of initial VEGF loading (n=4).
